# Impact of the conjugation of antibodies to the surfaces of polymer nanoparticles on the immune cell targeting abilities

**DOI:** 10.1186/s40580-021-00274-7

**Published:** 2021-08-16

**Authors:** Na Kyeong Lee, Chi-Pin James Wang, Jaesung Lim, Wooram Park, Ho-Keun Kwon, Se-Na Kim, Tae-Hyung Kim, Chun Gwon Park

**Affiliations:** 1 Department of Intelligent Precision Healthcare Convergence, Sungkyunkwan University, Suwon, Gyeonggi 16419 Republic of Korea; 2 Department of Biomedical Engineering, SKKU Institute for Convergence, Sungkyunkwan University (SKKU), Suwon, Gyeonggi 16419 Republic of Korea; 3 Department of Biomedical-Chemical Engineering, The Catholic University of Korea, Bucheon, Gyeonggi 14662 Republic of Korea; 4 Department of Microbiology and Immunology, Institute for Immunology and Immunological Diseases and Brain Korea 21 PLUS Project for Medical Sciences, Yonsei University College of Medicine, Seoul, 03722 Republic of Korea; 5 Institute of Medical and Biological Engineering, Medical Research Center, Seoul National University, Seoul, 03080 Republic of Korea; 6 School of Integrative Engineering, Chung-Ang University, 84, Heukseok-ro, Dongjak-gu, Seoul, 06974 Republic of Korea; 7 Biomedical Institute for Convergence at SKKU (BICS), Sungkyunkwan University, Suwon, Gyeonggi 16419 Republic of Korea; 8 Center for Neuroscience Imaging Research, Institute for Basic Science (IBS), Suwon, Gyeonggi 16419 Republic of Korea

**Keywords:** Target drug delivery, Nanoparticles, Antibody Conjugation, Maleimide-thiol reaction, Carbodiimide coupling

## Abstract

Antibodies have been widely used to provide targeting ability and to enhance bioactivity owing to their high specificity, availability, and diversity. Recent advances in biotechnology and nanotechnology permit site-specific engineering of antibodies and their conjugation to the surfaces of nanoparticles (NPs) in various orientations through chemical conjugations and physical adhesions. This study proposes the conjugation of poly(lactic-*co*-glycolic acid) (PLGA) NPs with antibodies by using two distinct methods, followed by a comparison between the cell-targeting efficiencies of both techniques. Full-length antibodies were conjugated to the PLGA-poly(ethylene glycol)-carboxylic acid (PLGA-PEG-COOH) NPs through the conventional carbodiimide coupling reaction, and f(ab′)_2_ antibody fragments were conjugated to the PLGA-poly(ethylene glycol)-maleimide(PLGA-PEG-Mal) NPs through interactions between the f(ab′)_2_ fragment thiol groups and the maleimide located on the nanoparticle surface. The results demonstrate that the PLGA nanoparticles conjugated with the f(ab′)_2_ antibody fragments had a higher targeting efficiency in vitro and in vivo than that of the PLGA nanoparticles conjugated with the full-length antibodies. The results of this study can be built upon to design a delivery technique for drugs through biocompatible nanoparticles.

**Supplementary Information:**

The online version contains supplementary material available at 10.1186/s40580-021-00274-7.

## Introduction

Antibodies have been widely used as active pharmaceutical ingredients, targeting ligands, and biosensing molecules in pharmaceutical and biomedical fields [1–3]. The rapid advancements in antibody development and large-scale production technologies have promoted the applications of antibodies in several fields [4, 5]. Antibodies are often chemically conjugated or physically adhered to the surfaces of biomaterial-based nanoparticles (NPs) and sensing platforms to ensure specific targeting or/and enhanced bioactivity due to their high specificity, remarkable diversity, and high availability. Such bioconjugates have already been successful in several biomedical fields, including biosensors [6–14] and targeted drug delivery [15–19]. Previous studies have demonstrated that NPs coated with antibodies have significantly enhanced drug delivery profiles in comparison to those of conventional drug carriers [20, 21]. In addition, modern breakthroughs in biotechnology and nanotechnology have enabled reliable site-specific engineering of antibodies and their conjugations on the surfaces of NPs in various orientations through distinct techniques.

The carbodiimide coupling reaction is a conventional technique that is widely used to conjugate antibodies to the surfaces of NPs [22–24]. This method involves activation of the carboxyl groups on the surface of the NPs, which can then be covalently conjugated to the amine groups of the antibodies. This technique is incredibly convenient for polymers functionalized with reactive carboxyl groups in their side chains [25–27]. However, several studies have reported that this reaction results in undesirable agglomeration and uncontrolled orientation of the conjugated antibodies, which reduces the conjugation efficiency of the antibodies [16, 19, 28, 29].

Another commonly used technique involves the conjugation of antibody fragments to the surfaces of NP surfaces instead of full-length antibodies. The antibody fragments were conjugated to the surfaces of the NPs by utilizing a chemical reaction between the maleimide and the thiol groups of the f(ab′)_2_ fragments [30]. Although the production of the f(ab′)_2_ fragments is expensive and conducted limitedly on certain IgG types [31, 32], the f(ab′)_2_ fragments generally exhibit higher specificity and stability in vivo due to the absence of various F_c_–mediated effector functions (e.g., antibody-dependent cellular cytotoxicity and phagocytosis) [33]. These superior characteristics of the f(ab′)_2_ fragments should provide enhanced *in vivo* profiles and target-binding abilities even when they are conjugated to the NPs. However, a direct head-to-head comparison between the targeting efficiency of NPs conjugated with full-length antibodies, and that of NPs conjugated with f(ab′)_2_ fragments has not been conducted.

Therefore, this study compares the cell-targeting efficiencies of the PLGA NPs conjugated with the f(ab′)_2_ fragments and full-length antibodies. The PLGA NPs were prepared by using conventional emulsification methods [34–36]. Full-length antibodies and f(ab′)_2_ fragments were chemically conjugated to their surfaces through conventional carbodiimide chemistry. The CD8a antibody was used as a model antibody in this study. CD8a is a cell surface glycoprotein and a marker of cytotoxic T lymphocytes [37]. It is easy to evaluate the cell-targeting abilities of CD8 T cells in vitro and in vivo because they are abundantly present in mouse models [38]. Full-length CD8a antibodies were conjugated to the PLGA NPs by using the carbodiimide coupling method to prepare Full-CD8a NPs. However, the f(ab′)_2_-CD8a NPs were prepared by conjugating the f(ab′)_2_ fragments to the PLGA NPs through maleimide reaction chemistry [30]. These antibody-conjugated NPs were first characterized to confirm the morphology, size, and complete conjugation of the antibodies. The number of antibodies conjugated to the surfaces of the NPs was quantified by using the BCA assay, followed by an evaluation of the antibody’s biding stability. The anti-CD8a antibodies were conjugated to fluorescent PLGA NPs by using different conjugation methods to observe their targeting efficiency towards CD8 T cells. After treating the two types of NPs, the immune cells targeted with the CD8a NPs were analyzed through flow cytometry in vitro and in vivo.

## Methods/experimental

### Preparation and characterizations of the conjugated NPs

PLGA NPs were prepared by using conventional emulsification methods [34–36]. The f(ab′)_2_-CD8a NPs were prepared by dissolving 22.5 mg of PLGA (M_w_: 10 000–15 000 Da, LG 50:50, PolySciTech, NH, USA) and 7.5 mg PLGA-poly(ethylene glycol)-maleimide (PLGA-PEG-Mal, M_w_: 10 000:5000 Da, LG 50:50, PolySciTech, NH, USA) in 1 mL of dichloromethane (DCM). The mixture was then poured into a 10 ml ice-cold solution of 2 % (w/v) poly(vinyl alcohol) (PVA). The resulting polymer solution was then sonicated for 10 min at a 20 % (140 W) amplitude according to the one sec-on and one sec-off sequence (Qsonica, CT, USA). The resulting emulsion was then stirred at room temperature for 4 h to completely evaporate the DCM. The PLGA-PEG-Mal NPs were collected through centrifugation at 17,000 rpm for 20 min. The collected NPs pellets were then washed with deionized (DI) water thrice through centrifugation at 17,000 rpm for 20 min.

Full-CD8a NPs were produced through a similar process; however, PLGA-poly(ethylene glycol)-COOH (PLGA-PEG-COOH, Mw: 10,000:5000 Da, LG:50:50, Ruixibiotech, Shannxi, China) (PLGA-PEG-COOH) was used instead of PLGA-PEG-Mal. The nanoparticles were traced through flow cytometry by adding 5 µg of DiIC18(5); 1,1′-dioctadecyl-3,3,3′,3′-tetramethylindodicarbocyanine, 4-chlorobenzenesulfonate salt (DiD, λEx/λEm: 644/663, Biotium, CA, USA) to 1 mg of NPs. The size, morphology, and zeta potential of the NPs were analyzed by using a Malvern Zetasizer Nano ZS system (Malvern Instruments, MA, USA), dynamic light scattering (DLS), and a JEM-7500 F (Akishima, Japan) scanning electron microscope (SEM).

### Conjugation of the antibodies to the NPs

The f(ab′)_2_-CD8a NPs, f(ab′)_2_ antibody fragments were chemically conjugated to the NPs by adopting a previously reported method [30]. Protease IdeS (Promega, WI, USA) was used to cleave full-length CD8a antibodies (Clone: 2.43; BioXcell, NH, USA) to the f(ab′)_2_ fragments. The f(ab′)_2_ fragments were then reduced with 2.5 µL of 10 mM DTT per 100 µg of antibodies to obtain free thiol groups in the hinge region. The free DTT was removed from the f(ab′)_2_ fragments by using 7 kDa MWCO desalting columns (Thermo Scientific, MA, USA) after reduction. This was followed by the addition of 5, 12.5, and 25 µg of antibodies to 1 mg of PLGA-PEG-Mal NPs (8 mg/mL) and incubation under shaking conditions (2 h, 25 °C). The BCA assay was used to quantify the number of antibodies conjugated to the surfaces of the NPs surface in comparison to the net number of antibodies initially added to the mixture.

The preparation of the full-CD8a NPs involved direct conjugation of the CD8a antibodies to the NPs through a carbodiimide coupling reaction [39]. *N*-hydroxysulfosuccinimide (NHS, 200 mM, 240 µL) and 1-ethyl-3-[3-dimethylaminopropyl] carbodiimide (EDC, 200 mM, 24 µL) were added to the PLGA-PEG-COOH NPs (5 mg/mL, 1mL) to activate the carboxyl group of the NPs. The activated PLGA-PEG-COOH NPs were washed with 1 × PBS thrice. The antibodies were added to the PLGA-PEG-COOH NPs and coupled at a ratio of 25 or 50 µg of antibodies per 1 mg of NPs. The duration of the NPs incubated with NHS/EDC for activation or antibody coupling are listed in Tables 1 and 2. The BCA assay was used to confirm the conjugation of antibodies to NPs. A standard reference curve was first established by adding the same amount of bare NPs to each well and then adding a different amount of antibodies to each well. The BCA assay data of antibody-conjugated NPs were then carefully analyzed upon the standard curve.

### In vitro binding stability of the antibodies conjugated to the NPs

The binding stability of the antibodies conjugated to f(ab′)_2_-CD8a and full-CD8a NPs was evaluated by dispersing 4 mg/mL of the antibody-conjugated NPs in Dulbecco’s phosphate-buffered saline (DPBS) and incubating in a shaker at 25 ℃. The solution was centrifuged at 17,000 rpm for 20 min after incubating the mixture for a fixed duration (1, 3, 5, and 7 days). The resulting supernatant was analyzed for its antibody content by using the BCA assay. The stability of the antibody conjugation was evaluated by comparing the initial number of antibodies conjugated to the NPs and the number of antibodies that detached from the NPs after 1, 3, and 5 days of incubation.

### Animals

Experiments were conducted on animals in accordance with the protocols approved by the Institutional Animal Care and Use Committee (IACUC) of Sungkyunkwan University College of Medicine (IACUC No. SKKU IACUC2021-01-33-3). Six- to seven-week-old C57BL/6 male mice (18–20 g) were obtained from Orient Bio (Seongnam, Korea). The mice were provided an acclimatization period of seven days before commencing the experiment. Five animals were housed in each cage at an automatically controlled temperature and humidity of 20–26 °C and 40–60 %, respectively, with a 12:12 h light/dark cycle. The mice were fed a standard rodent pellet diet and supplied with water ad libitum.

### Evaluation of the immune cell-targeting ability of the NPs in vitro

The in vitro targeting efficiency of the NPs was investigated by using mouse splenocytes. The spleen of a C57BL/6 mouse (6-week-old) was first extracted. It was triturated on a 70 μm cell strainer by using the rear end of the plunger of a 1 mL syringe. The gathered cells were then centrifuged at 1500 rpm for 5 min. The pellets were then re-suspended in 5 mL of ACK lysis buffer (Gibco, MA, USA) for 3 min to eliminate red blood cells. The splenocytes were collected after centrifuging the cells at 1500 rpm for 5 min. DiD-loaded NPs were co-incubated with mouse splenocytes (2 × 10^6^ cells) in a 96 well plate while they were suspended in DPBS. The cells were treated with different concentrations of DiD-loaded NPs for a duration that varied from 30 to 180 min. The cells were washed in DPBS thrice after co-incubation. Our target immune cells were then assessed through flow cytometry. The antibodies used to gate the target immune cells include Zombie Violet (Biolegend, CA, USA) for Live/Dead staining, PE-TCR β chain (Clone: H57-597; Biolegend, CA, USA), PE/Cy7-CD4 (Clone: GK 1.5; Biolegend, CA, USA), and FITC-CD8a (Clone: 53−6.7; Biolegend, CA, USA).

### Evaluation of the immune cell-targeting efficiency of the NPs in vivo

DiD was added at 10 µg per 2 mg of f(ab′)_2_-CD8a NPs, followed by injecting the NPs into the tail veins of C57BL/6 mice. The blood and spleen samples of the mice were harvested for subsequent analysis after euthanizing them at preset time points (1, 3, or 6 h). The CD8 T cells were then assessed for DiD signals through flow cytometry to evaluate the NPs’ in vivo cell-targeting efficiency.

### Statistical analyses

Statistical analyses were conducted by using 2-way ANOVA, and 1-way ANOVA with Sidak’s multiple comparisons test through the *Graphpad Prism* software. The data obtained in this study were formatted as “mean ± standard error of the mean” or “mean ± standard deviation (SD)” with a significance set of P < 0.05. In addition, the P-values and detailed information for each experiment was provided in each Figures’ legends.

## Results and discussion

### Preparation and characterization of the PLGA-PEG-COOH NPs and PLGA-PEG-Mal NPs

**Scheme 1:**
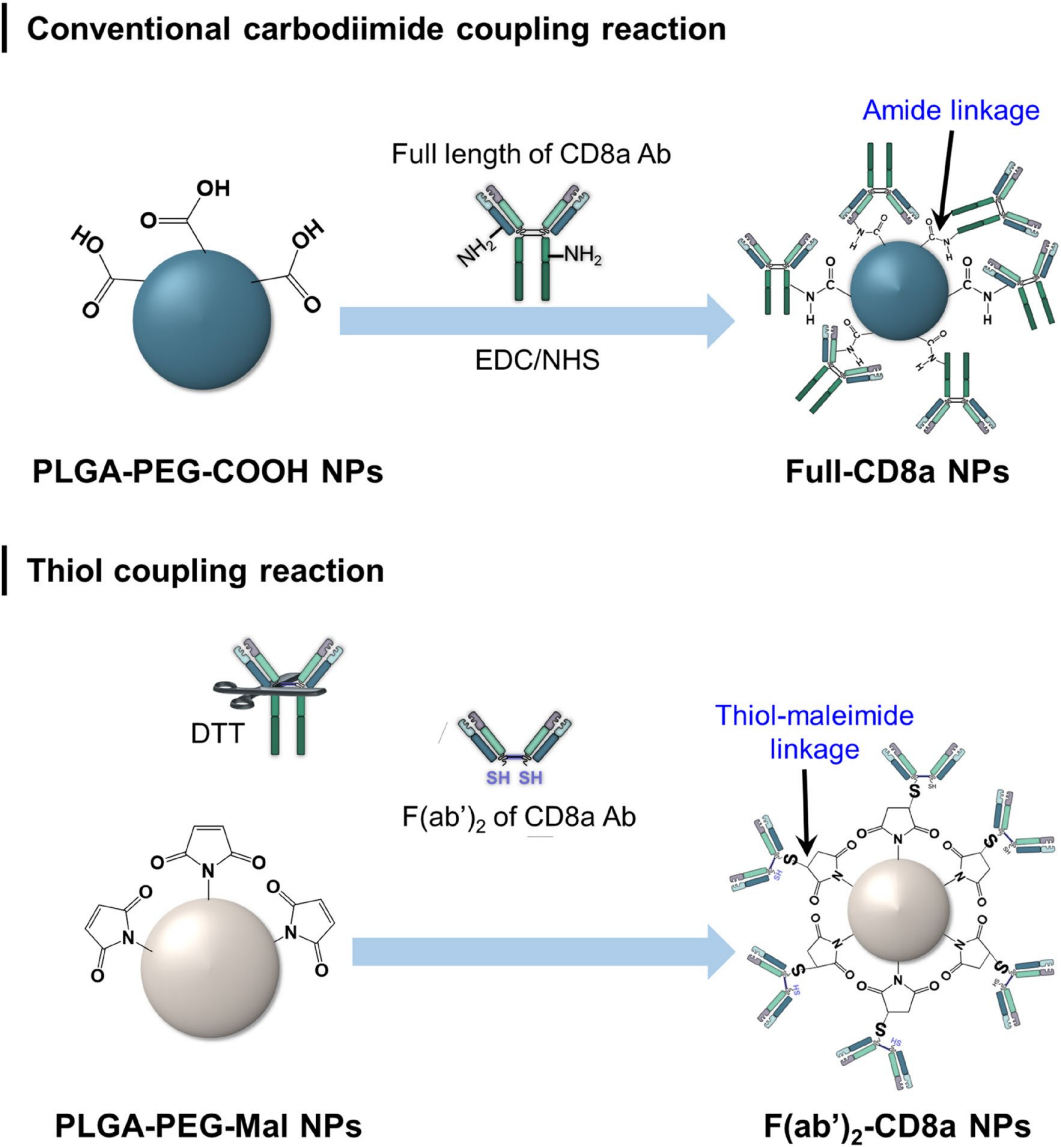
Schematic of the synthetic route of the antibody-conjugated NPs. (Upper) Synthesis of the full-CD8a NPs. The EDC activates the COOH group of the PLGA NPs, and the NHS subsequently forms a stabilized amine-reactive sulfo-NHS ester. This allows the NH_2_ group of the antibody to bind with the amine-reactive sulfo-NHS ester on the NP surface. (lower) Synthesis of the f(ab′)_2_-NPs. An antibody was first cleaved by using IdeS. It was then reduced by DTT to obtain a free thiol group in its hinge region. The free thiol group binds with the Mal located on the NP surface

The PLGA NPs were prepared through a single emulsion method [34–36], and the CD8a antibodies were conjugated to the surfaces of the NPs through carbodiimide [22] and maleimide techniques [30] (Scheme 1; Additional file 1: Fig S1). The f(ab′)_2_-CD8a NPs were synthesized by first cleaving full-length antibodies to f(ab′)_2_ and fc fragments using the protease IdeS. The presence of characteristic bands confirmed the successful fragmentation of anti-CD8a during a non-reducing SDS-PAGE analysis (Additional file 1: Fig. S3). DTT was then used to create a free thiol group in the hinge region of the f(ab′)_2_ fragments.

**Fig. 1:**
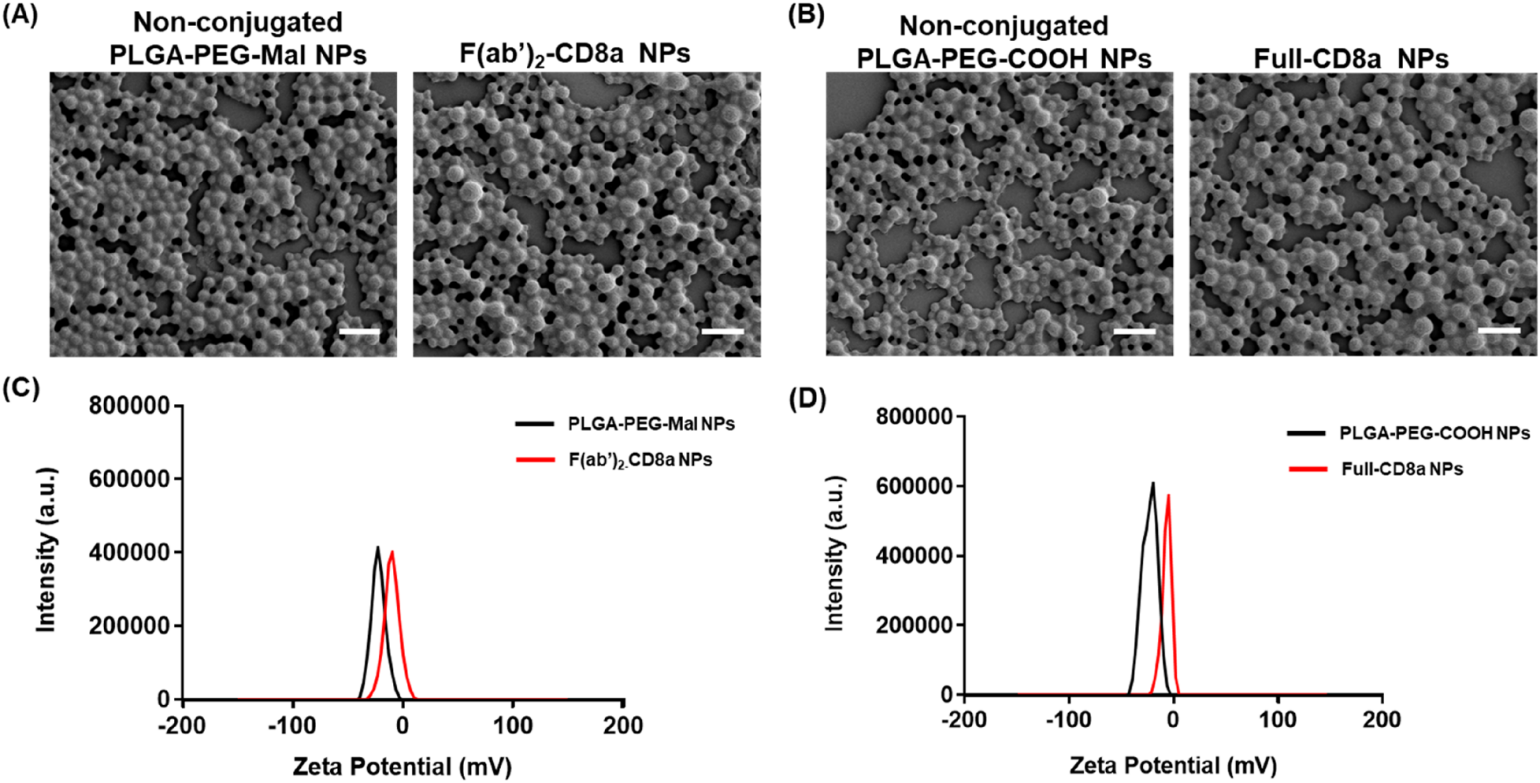
Morphological and zeta potential analysis of the PLGA NPs through scanning electron microscopy (SEM) and dynamic light scattering (DLS). **A** SEM image of non-conjugated PLGA-PEG-COOH NPs and Full-CD8a NPs. **B** SEM image of non-conjugated PLGA-PEG-Mal NPs and f(ab′)_2_-CD8a NPs (Scale bar = 0.5 μm). **C** Zeta potential of PLGA-PEG-Mal NPs and f(ab′)_2_-CD8a NPs. **D** Zeta potential of PLGA-PEG-COOH NPs and Full-CD8a NPs

The morphologies and sizes of both NPs were evaluated with SEM and DLS. The average sizes of the non-conjugated-PLGA-PEG-Mal NPs and f(ab′)_2_-CD8a NPs were measured with DLS to be 193 and 201 nm, respectively (Additional file 1: Fig. S2A). Similarly, the average sizes of the non-conjugated-PLGA-PEG-COOH NPs and full-CD8a NPs were 195 nm and 205 nm, respectively (Additional file 1: Fig. S2B). These results are consistent with previous hypotheses that state that the sizes of the PLGA NPs are primarily determined by their polymer concentrations and weights instead of their surface functional groups [40, 41]. Since the size of a full-length antibody is approximately 10 nm [29], the slight increase in the size of the NP observed after antibody conjugation can be attributed to the presence of the conjugated surface antibodies. The absence of the fc region can also explain why the f(ab′)_2_-CD8a NPs are slightly smaller than full-CD8a NPs. Further, SEM images of the full-CD8a NPs and f(ab′)_2_-CD8a NPs confirmed that there were no significant variations between the spherical morphologies and sizes of the NPs before and after antibody conjugation (Fig. 1A, B). The zeta potentials of PLGA-PEG-Mal NPs and f(ab′)_2_-CD8a NPs were − 22.3 mV and − 6.78 mV, respectively (Fig. 1C). The zeta potentials for PLGA-PEG-COOH NPs and full-CD8a NPs were each − 22.9 mV and − 6.49 mV (Fig. 1D). In general, the surface charges of all PLGA NPs were negative [42, 43]. The less negative zeta potential of the antibody-conjugated NPs can therefore be explained by the presence of antibodies on the NPs’ surface.

### Measurement of the number of antibodies conjugated to the surfaces of the NPs

The feed number of the antibodies and the reaction time were varied to study the antibody conjugation efficiency. The NPs were prepared under different conditions to observe the impact of different parameters on antibody conjugation and to different models of NPs for further applications.

The duration of the COOH activation in full-CD8a NPs, which was induced by the carbodiimide reaction, and the coupling duration were varied. The net amount of anti-CD8a (25–50 µg) added to the coupling reaction was also varied. It was observed that the increased activation time, coupling time, and initial number of antibodies increased the final number of antibodies conjugated to the NPs (Tables 1, 2). It was also observed that an increment in initial concentration of the f(ab′)_2_ fragments during their reaction with the PLGA-PEG-Mal NPs significantly increased the number of antibodies conjugated to the surfaces of the NPs (Table 3).

**Table 1:** The amounts of anti-CD8a Ab conjugated to the PLGA-PEG-COOH NPs. The amounts (µg) of antibodies conjugated to 1 mg of the PLGA-PEG-COOH NPs when antibodies were initially added at 25 µg per 1 mg of NPs was measured by BCA assay (n = 3, mean ± SD)

Coupling time (h)		Activation time (h)		
		3	6	10
3		1.74 ± 0.39	3.48 ± 0.44	2.90 ± 0.59
6		2.42 ± 0.72	3.61 ± 0.50	3.41 ± 1.00
10		3.36 ± 0.40	3.86 ± 0.32	5.25 ± 0.30

**Table 2:** The amounts of anti-CD8a Ab conjugated to the PLGA-PEG-COOH NPs. The amounts (µg) of antibodies conjugated to 1 mg of the PLGA-PEG-COOH NPs when antibodies were initially added at 50 µg per 1 mg of NPs was measured by BCA assay (n = 3, mean ± SD)

Coupling time (h)		Activation time (h)		
		3	6	10
3		3.56 ± 0.58	4.82 ± 0.29	4.34 ± 0.66
6		5.28 ± 0.49	4.82 ± 0.50	6.31 ± 0.35
10		5.28 ± 0.75	5.55 ± 0.27	7.32 ± 0.95

**Table 3:** The amounts of anti-CD8a Ab conjugated to the PLGA-PEG-Mal NPs. The amounts of antibodies conjugated to 1 mg of PLGA-PEG-Mal NPs when different numbers of antibodies were added initially was measured by BCA assay (n = 3, mean ± SD)

	Feed amount of Anti-CD8a Ab (per 1 mg NPs, µg)		
	5	12.5	25
Amounts of conjugated Anti-CD8a Abs (per 1 mg NPs, µg)	1.32 ± 0.36	5.19 ± 0.16	11.80 ± 0.01

Three types of f(ab′)_2_-CD8a NPs, namely f(ab′)_2_-CD8a NPs-low, f(ab′_2_-CD8a NPs-medium, and f(ab′)_2_-CD8a NPs-high with 1.32 ± 0.36, 5.19 ± 0.16, and 11.80 ± 0.01 µg/mg of coated antibody fragments, were selected for additional in vitro and in vivo experiments. We also selected three full-CD8a NPs with corresponding numbers of conjugated antibodies (i.e., the numbers of coated full-length antibodies for the full-CD8a NPs-low, full-CD8a NPs-medium, and full-CD8a NPs-high variants were 1.74 ± 0.39, 5.28 ± 0.49, and 7.32 ± 0.95 µg/mg, respectively).

**Fig. 2:**
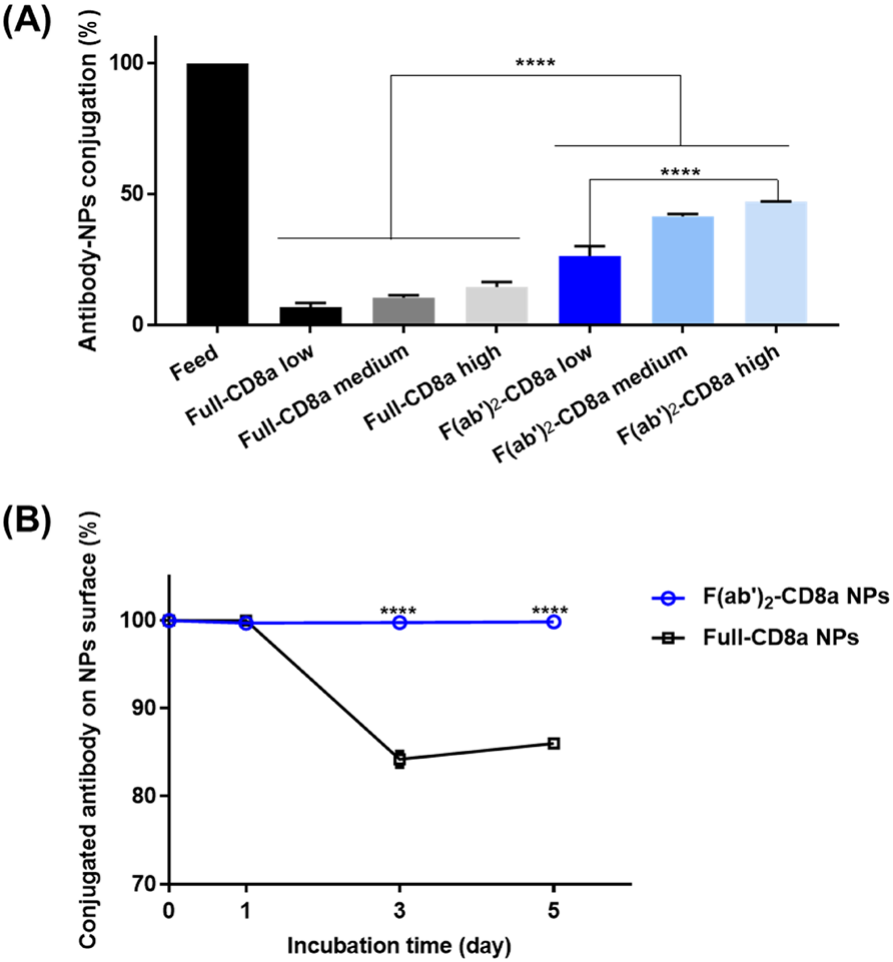
Conjugation efficiency and stability of the antibodies to the surface of NPs. **A** Conjugation efficiency of each NPs compared with added antibody. Data were presented as the mean ± SD (n = 3, ****P < 0.0001). **B** The binding stability of the antibodies conjugated to the NPs is determined by the change in the number of antibodies bound to the NP surface over the incubation period (25 ℃, shaking condition). Data were presented as the mean ± SD (n = 2, ****P < 0.0001)

The efficiency of the antibody conjugation to the surfaces of the NPs was calculated by setting the total number of antibodies added to the particles to 100 % (Fig. 2A). The efficiencies of the full-CD8a NPs-low, medium, and high variants were 6.96 %, 10.55 %, and 14.64 %, respectively. Similarly, the efficiencies of the f(ab′)_2_-CD8a NPs-low, medium, and high variants were 26.44 %, 41.53 %, and 47.19 %, respectively. This indicates that the conjugation of the f(ab′)_2_ fragments to the surface of the PLGA-PEG-Mal NPs was more efficient than that of the full-length antibodies to the PLGA-PEG-COOH NPs.

### In vitro binding stability of the antibodies conjugated to the NPs

The in vitro binding stability of the antibodies conjugated to the NPs was calculated by measuring the number of antibodies remaining conjugated to the nanoparticles after incubating the NPs in DPBS. The BCA assay was utilized to measure the remaining number of antibodies conjugated to the NPs and compared it with the initial number of conjugated antibodies (Fig. 2B). The f(ab′)_2_-CD8a NPs demonstrated excellent stability under shaking conditions, with 99 % of its initial antibodies remaining until day 5. However, approximately 16 % of the conjugated antibodies of the full-CD8a NPs were reduced within five days, suggesting that the chemical bonding stability of carbodiimide coupling in the full-CD8a NPs may be weaker than the maleimide coupling in the f(ab′)_2_-CD8a NPs. Thus, the stability of the f(ab′)_2_-CD8a NPs was superior to that of the full-CD8a NPs under prolonged periods of incubation. This confirms that the maleimide coupling procedure has a much longer shelf life than that of carbodiimide chemistry.

### Immune cell targeting efficiency of the NPs in vitro

The immune cell-targeting efficiency of the antibody-conjugated NPs was analyzed by treating the mouse splenocytes with 10 µg of DiD-loaded NPs for 10 min. The CD8 T and CD4 T cells were then analyzed for DiD signals through flow cytometry. The CD8 and CD4 T cells were primarily gate based on the expressions of the TCRβ, CD4, and CD8a markers on the surface of each cell (Additional file 1: Fig. S4A). The antibody-conjugated NPs interacted extensively with the CD8 T cells in comparison to the control NPs that were not conjugated with antibodies, suggesting that the antibody-conjugated NPs could successfully target the CD8 T cells.

**Fig. 3:**
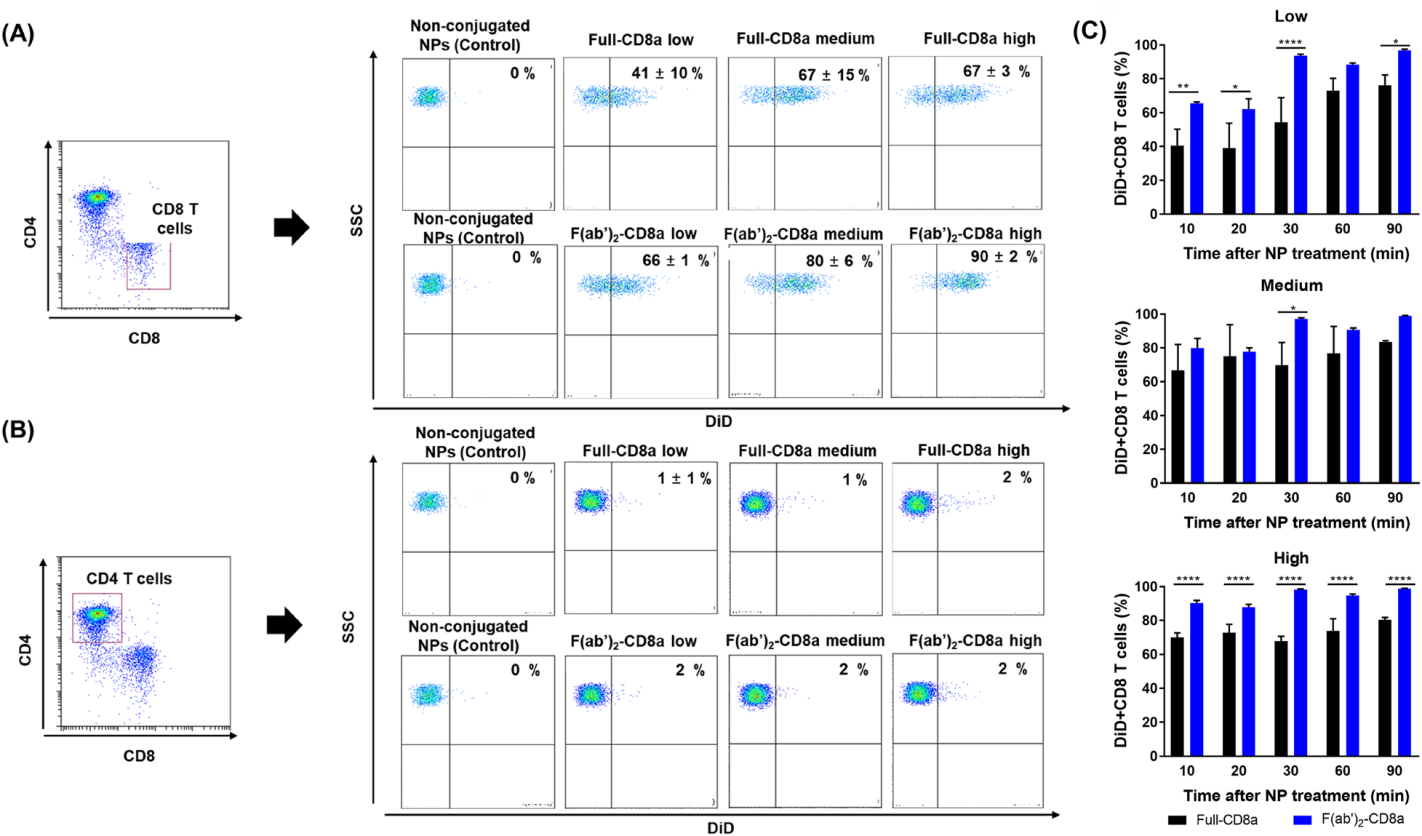
T cell targeting efficiency of the CD8a-conjugated NPs in vitro. **A** Binding of the anti-CD8a conjugated NPs to the CD8a T cells. The mouse splenocytes were treated with the NPs for 10 min after loading the NPs with DiD and assessed through flow cytometry. **B** Observed DiD signal in the CD4 T cells that confirms the minimal non-specific binding of the anti-CD8a conjugated NPs, and (C) Comparison between the populations of the DiD-expressing CD8 T cells induced by the full-CD8a NPs and f(ab′)_2_-CD8a NPs at different instants. Data were presented as the mean ± SD (n = 3, *P = 0.0332, **P = 0.0021, ***P = 0.0002, ****P < 0.0001)

The full-CD8a NPs-low and f(ab′)_2_-CD8a NPs-low variants and the full-CD8a NPs-medium and f(ab′)_2_-CD8a NPs-medium variants are pairs with similar numbers of surface antibodies. On the basis of the abovementioned statement (Fig. 3A), a direct comparison between the pairs’ binding efficiencies was conducted, which indicated that the average increment in the DiD + CD8 T cell population induced by the f(ab′)_2_-CD8a NPs-low variant (66 %) was 1.61 times that of the full-CD8a NPs-low variant (41 %). Similarly, the average increment in the DiD + CD8 T cell population induced by the f(ab′)_2_-CD8a NPs-medium variant (80 %) was 1.2 times that of the full-CD8a NPs-medium variant (67 %). Further, the average increment in the DiD + CD8 T cell population induced by the f(ab′)_2_-CD8a NPs-high variant (90 %) was 1.3 times that of the full-CD8a NPs-high variant (67 %). This indicates that the f(ab′)_2_-CD8a NPs have a higher targeting efficiency than that of the full-CD8a NPs. The non-specific binding of the NPs was evaluated by analyzing the DiD signals obtained from the CD4 T cells because both NPs were not designed to target them (Fig. 3B). The minimal presence of the DiD + CD4 T cells in all NP groups was found to be similar to that of the control NPs. The non-specific binding nature of the NPs at different instants is shown in Additional file 1: Fig. S4B. These results suggest that the antibody-conjugated NPs can successfully target specific cells without undesired non-specific bindings, irrespective of their conjugation chemistry. It provides an experimental insight into the possibility of utilizing of antibody-conjugated NPs as effective platforms of target delivery.

The mouse splenocytes were co-incubated with NPs for a period greater than 10 min (Fig. 3C). The prolonged incubation increased the population of the DiD + CD8 T cells. The f(ab′)_2_-CD8a NPs-low, f(ab′)_2_-CD8a NPs-medium, and f(ab′)_2_-CD8a NPs-high variants attained binding efficiencies of nearly 100 % after 90 min of co-incubation. However, the binding efficiencies of the full-CD8a NPs-low, full-CD8a NPs-medium, and full-CD8a NPs-high variants were limited to approximately 80 %. These results demonstrate that the binding efficiencies of the f(ab′)_2_-CD8a NPs are superior to those of the full-CD8a NPs *in vitro*. The population of the DiD + CD8 T cells increased at all instants for all variants after increasing the amount of DiD loaded NPs to 20 µg (Additional file 1: Fig. S4C). Thus, the in vitro data indicate that the f(ab′)_2_-CD8a NPs can bind to the CD8 T cells more quickly and efficiently than the full-CD8a NPs.

The difference between the binding efficiencies of the f(ab′)_2_-CD8a NPs and full-CD8a NPs can be attributed to the random orientation of the full-length antibodies conjugated to the full-CD8a NPs. This study utilized the carbodiimide coupling method to conjugate full-length antibodies to the surfaces of the fc region. Previous studies have reported that the experimental condition frequently required during the implementation of the conventional carbodiimide coupling methods is also capable of inducing active conjugation through the antibody’s f(ab′)_2_ region, which possesses highly reactive amine moieties [16]. The full-length antibodies conjugated to the full-CD8a NPs may not be oriented appropriately due to the presence of competing regions thereby, limiting their antigen-binding efficiency. However, this interpretation requires further imaging of the surfaces of the NP through an ultra-high resolution SEM for proper verification.

### Immune cells targeting efficiency of the NPs in vivo

**Scheme 2:**
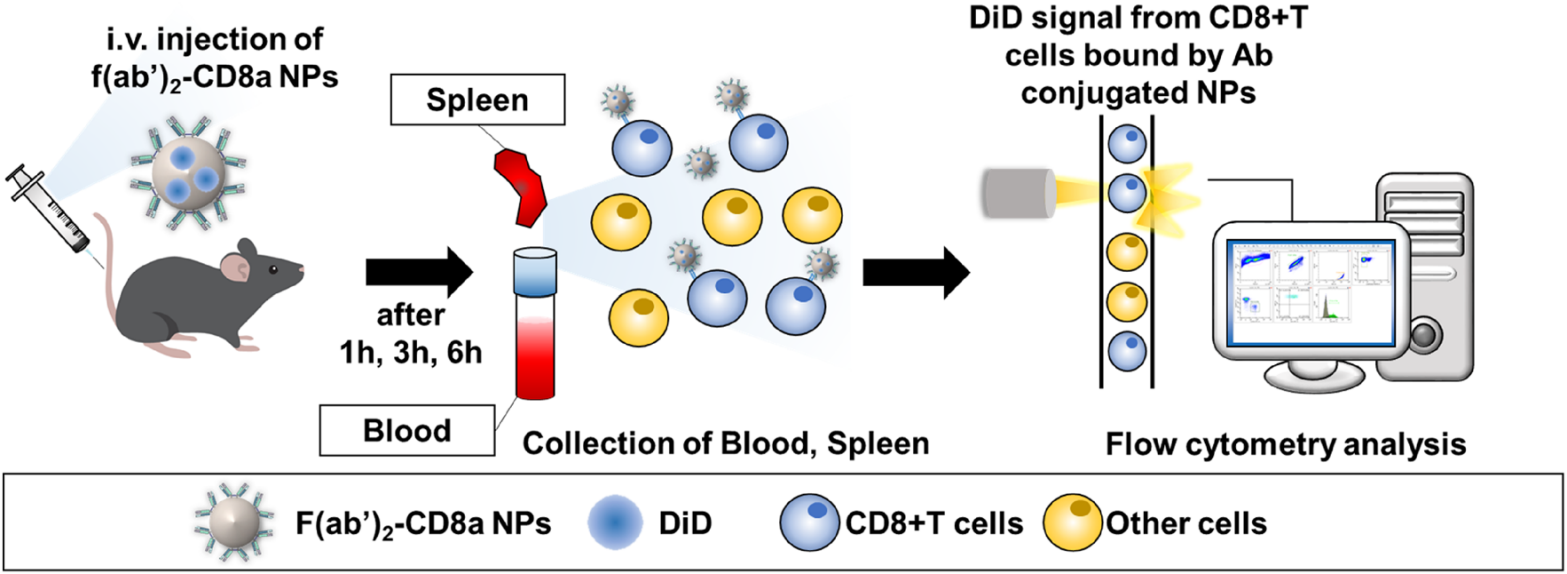
Analysis of the T cell targeting efficiency of the f(ab′)_2_-CD8a NPs in vivo. The F(ab′)_2_-CD8a NPs were administered to a mice model through an intravenous injection. The spleen and blood from the injected mouse were collected after 1–6 h and analyzed through flow cytometry to confirm the existence of the DiD + CD8 T cells

**Fig. 4:**
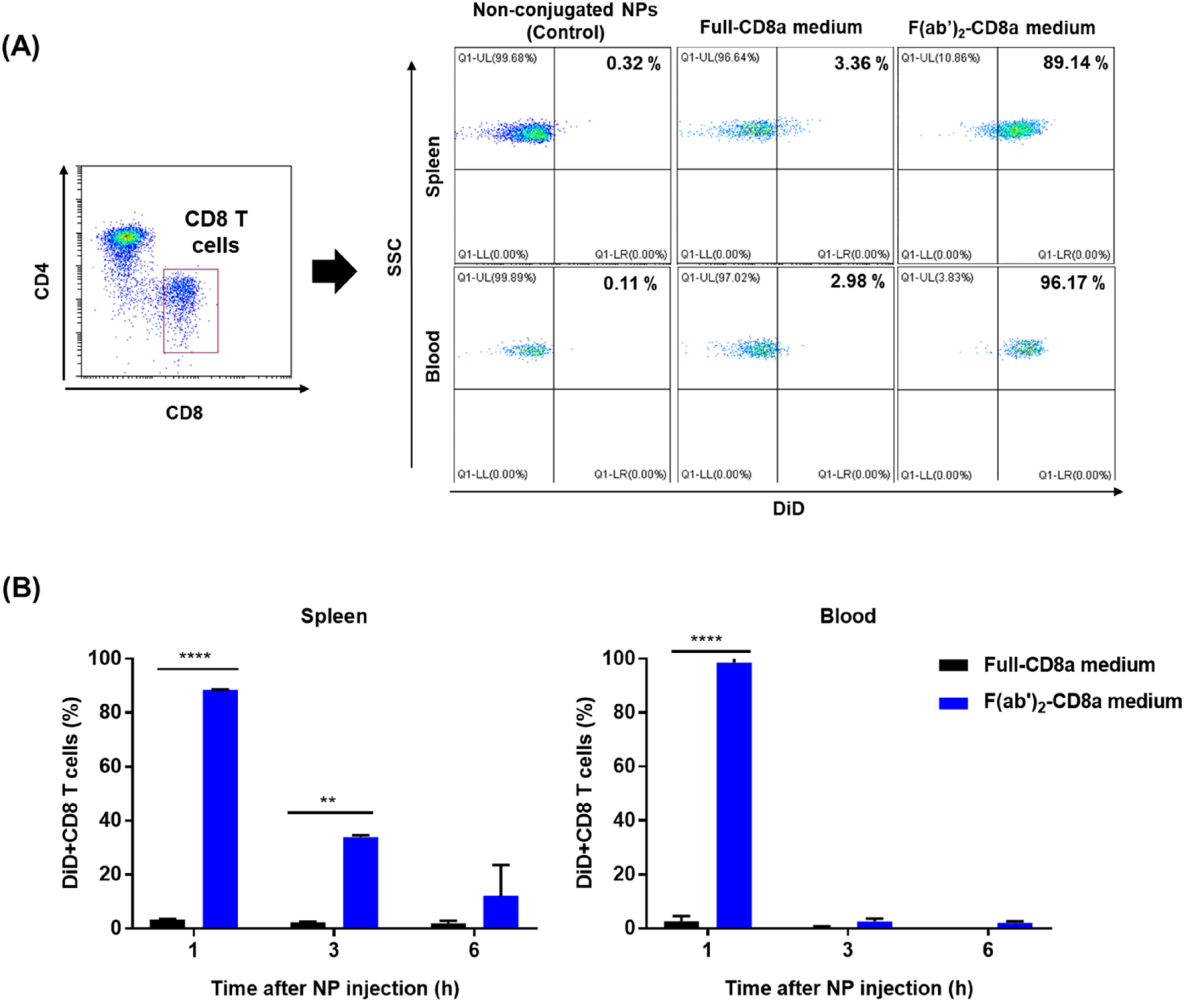
CD8a T cell targeting efficiency of the f(ab′)_2_-CD8a NPs-medium in vivo. **A** Population of the DiD + CD8 T cells after 1 h of circulation. The NPs are administered through IV injection and assessed through flow cytometry. **B** Clearance of the administered NPs observed by the variation of the population of the DiD + CD8 T cells across prolonged circulation. Data were presented as the mean ± standard error of the mean (n = 3, **P = 0.0021, ****P < 0.0001)

Based on the stability of conjugated antibodies and in vitro binding efficiency, the f(ab′)_2_-CD8a NPs-medium was selected for further *in vivo* CD8 T cell targeting efficiency test. The f(ab′)_2_-CD8a NPs-medium was injected through the IV route. The mouse spleen and blood samples were taken at different instants (1, 3, and 6 h) and analyzed through flow cytometry (Scheme 2). The population of the DiD + CD8 T cells observed in the spleen was 88.6 and 98.59 % in the blood of the mice injected with f(ab′)_2_-CD8a medium NPs (Fig. 4A).

A rapid reduction in the population of the DiD + CD8 T cells was observed after one hour of in vivo circulation, irrespective of the administered NPs (Fig. 4B). This reduction in the DiD signals suggests that the f(ab′)_2_-CD8a-medium experienced active systematic clearance after 3 and 6 h of circulation in the spleen and blood, respectively.

## Conclusions

We prepared and compared the targeting efficiencies of full-CD8a NPs and f(ab′)_2_-CD8a NPs in this study, both in vivo and in vitro. The SEM and DLS data showed that the variations between the sizes and morphologies of both NPs are minimal. The number and stability of the antibodies conjugated to the NP surfaces of the f(ab′)_2_-CD8a NPs were higher than those of the NP surfaces of the full-CD8a NPs. The in vitro data confirmed that the cell-binding ability of the f(ab′)_2_-CD8a NPs was more rapid and efficient than that of the full-CD8a NPs. And also, the f(ab′)_2_ -CD8a NPs showed successfully in vivo targeting ability through the flow cytometry analysis of the spleen and blood in the mouse injected with the f(ab′)_2_-CD8a NPs. The results confirm that the f(ab′)_2_-CD8a NPs are highly capable of minimizing non-specific binding while exhibiting excellent cell-targeting efficiency, thereby confirming its enormous potential as a future target delivery platform in comparison to the full-CD8a NPs. Therefore, the proposed new type of antibody conjugation method combined with biocompatible polymeric NPs can be a promising candidate that might overcome the limitations of the current NP-based target delivery platform using complete antibodies.

## Supplementary Information

**Additional file 1: Figure S1. **Synthesisof the antibody-conjugated nanoparticles (NPs). **Figure S2**. Particlesize analysis of the antibody-conjugated nanoparticles (NPs). **Figure S3**. F(ab′)_2_ fragments of theanti-CD8a antibody. **Figure S4**. T cell targeting efficiency of theCD8a-conjugated nanoparticles (NPs) in vitro. 

## Data Availability

The datasets used and/or analyzed during the current study are available from the corresponding author after submitting a reasonable request.

## Abbreviations

NPs: Nanoparticles
PLGA: Poly(lactic-*co*-glycolic acid)
PLGA-PEG-Mal: PLGA-poly(ethylene glycol)-maleimide
PLGA-PEG-COOH: PLGA-poly(ethylene glycol)-carboxylic acid
NHS: *N*-hydroxysulfosuccinimide
EDC: 1-Ethyl-3-[3-dimethylaminopropyl] carbodiimide
F(ab′)_2_-CD8a NPs: Dynamic light scattering
SEM: Scanning electron microscope
DiD: DiIC18(5); 1,1′-dioctadecyl-3,3,3′,3′- tetramethylindodicarbocyanine, 4-chlorobenzenesulfonate salt
DPBS: Dulbecco's phosphate-buffered saline
PVA: Poly (vinyl alcohol)
DI: Deionized
Full-CD8a NPs: PLGA-PEG-COOH NPs conjugated with the full length of the anti-CD8a antibody
F(ab′)_2_-CD8a NPs: PLGA-PEG-Mal NPs conjugated with the f(ab′)_2_ fragments of the anti-CD8a antibody
