# Supplementary material for: Impact of the conjugation of antibodies to the surfaces of polymer nanoparticles on the immune cell targeting abilities
Source: Nano Converg. 2021 Aug 16;8:24. doi: 10.1186/s40580-021-00274-7 (PMC8368787; doi:10.1186/s40580-021-00274-7)
Supplement: Supplementary file 1 — Additional file 1: Figure S1. Synthesisof the antibody-conjugated nanoparticles (NPs). Figure S2. Particlesize analysis of the antibody-conjugated nanoparticles (NPs). Figure S3. F(ab′)2 fragments of theanti-CD8a antibody. Figure S4. T cell targeting efficiency of theCD8a-conjugated nanoparticles (NPs) in vitro. [file 40580_2021_274_MOESM1_ESM.docx]

*Supporting Information*

**Impact of the Conjugation of Antibodies to the Surfaces of Polymer Nanoparticles on the Immune Cell Targeting Abilities**

*Na Kyeong Lee^1^, Chi-Pin James Wang^2^, Jaesung Lim^1^, Wooram Park^3^, Ho-Keun Kwon^4^, Se-Na Kim^5^, Tae Hyung Kim^6, *^, and Chun Gwon Park^1,2,7,8, *^*

^1^Department of Intelligent Precision Healthcare Convergence, Sungkyunkwan University, Suwon, Gyeonggi 16419, Republic of Korea

^2^Department of Biomedical Engineering, SKKU Institute for Convergence, Sungkyunkwan University (SKKU), Suwon, Gyeonggi 16419 Republic of Korea

^3^Department of Biomedical-Chemical Engineering, The Catholic University of Korea, Bucheon, Gyeonggi 14662, Republic of Korea

^4^Department of Microbiology and Immunology, Institute for Immunology and Immunological Diseases and Brain Korea 21 PLUS Project for Medical Sciences, Yonsei University College of Medicine, Seoul, 03722, Republic of Korea

^5^Institute of Medical & Biological Engineering, Medical Research Center, Seoul National University, Seoul 03080, Republic of Korea

^6^School of Integrative Engineering, Chung-Ang University, Seoul 06974, Republic of Korea

^7^Biomedical Institute for Convergence at SKKU (BICS), Sungkyunkwan University, Suwon, Gyeonggi 16419, Republic of Korea

^8^Center for Neuroscience Imaging Research, Institute for Basic Science (IBS), Suwon, Gyeonggi 16419, Republic of Korea

***Corresponding authors**

[thkim0512@cau.ac.kr](mailto:thkim0512@cau.ac.kr) (T. H. Kim) and [chunpark@skku.edu](mailto:chunpark@skku.edu) (C.G. Park)


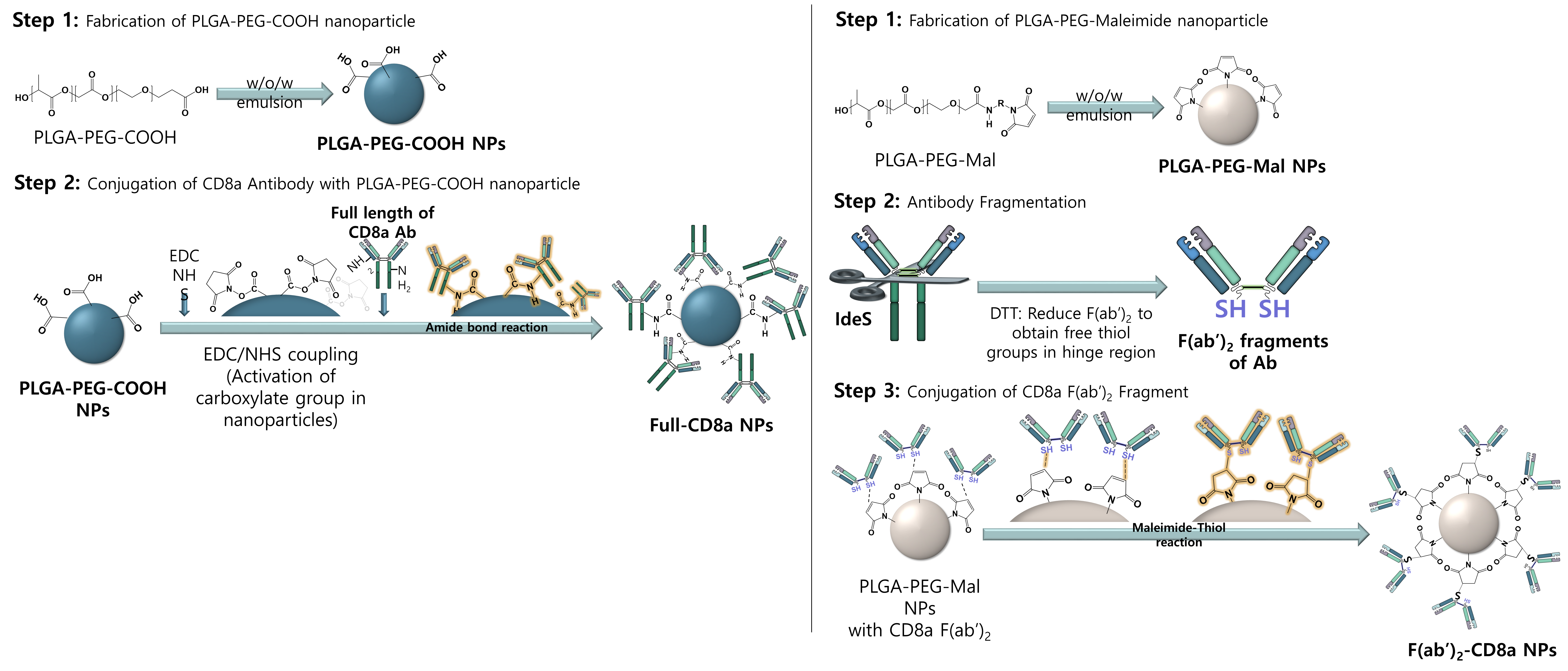


Figure S1. Synthesis of the antibody-conjugated nanoparticles (NPs).


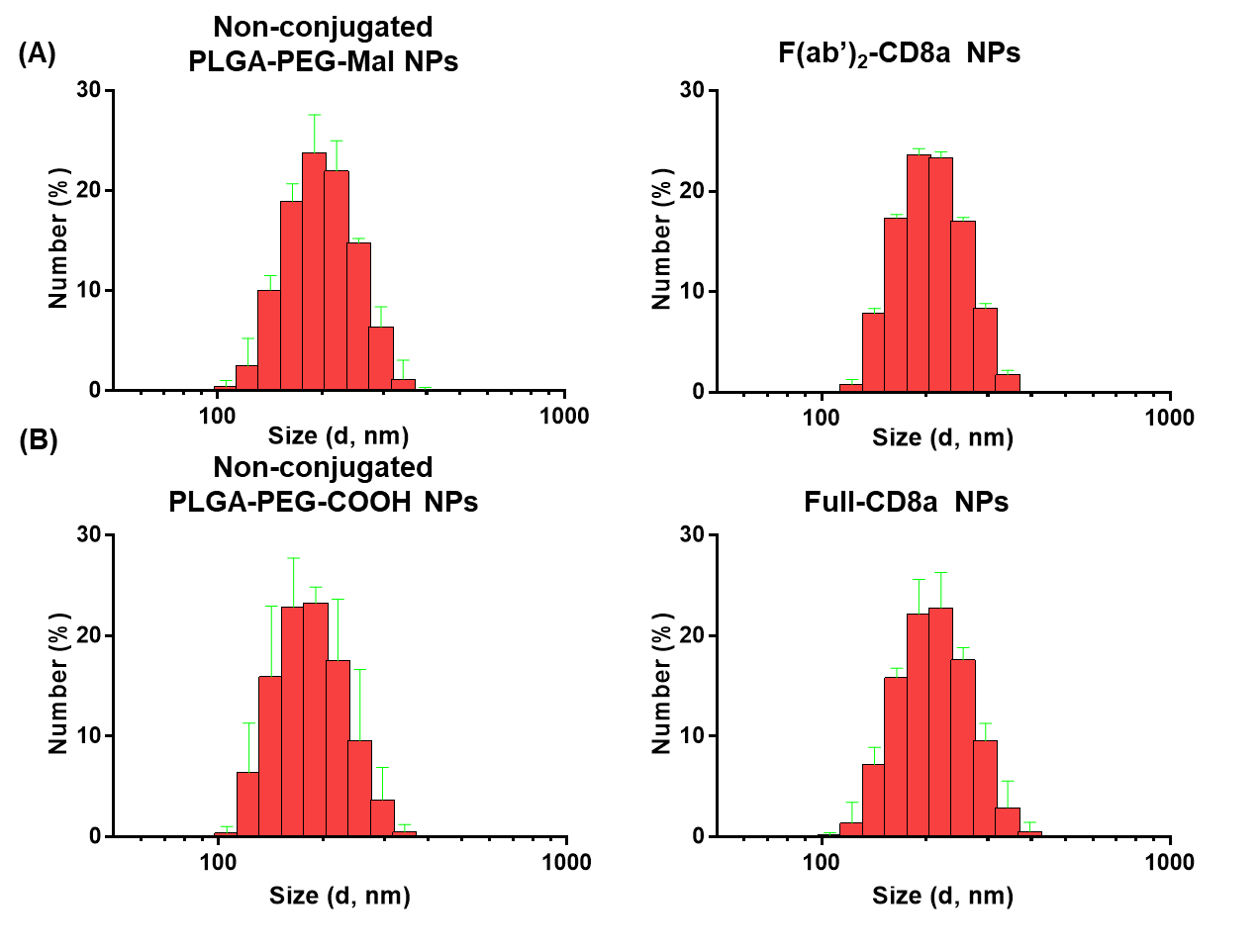


Figure S2. Particle size analysis of the antibody-conjugated nanoparticles (NPs). Size distributions of the NPs conjugated with the (A) f(ab’)_2_ antibody fragments (F(ab’)_2_-CD8a NPs) and the (B) full-length CD8 antibodies (Full-CD8 NPs) measured through dynamic light scattering (DLS). Data were presented as the mean ± SD (n=3).


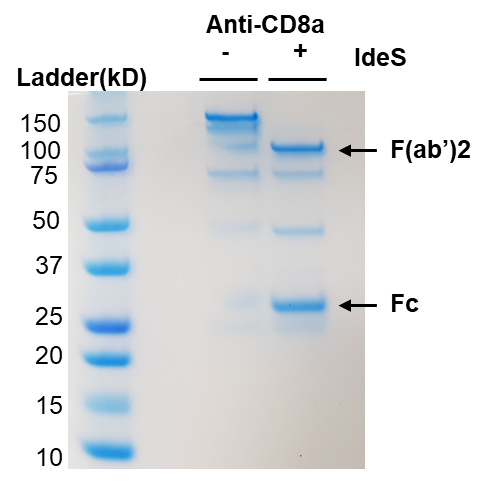


Figure S3. F(ab’)_2_ fragments of the anti-CD8a antibody. A non-reducing SDS-PAGE gel stained with Coomassie Brilliant Blue shows the successful cleavages of the f(ab’)_2_ and fc fragments through the IdeS protease.


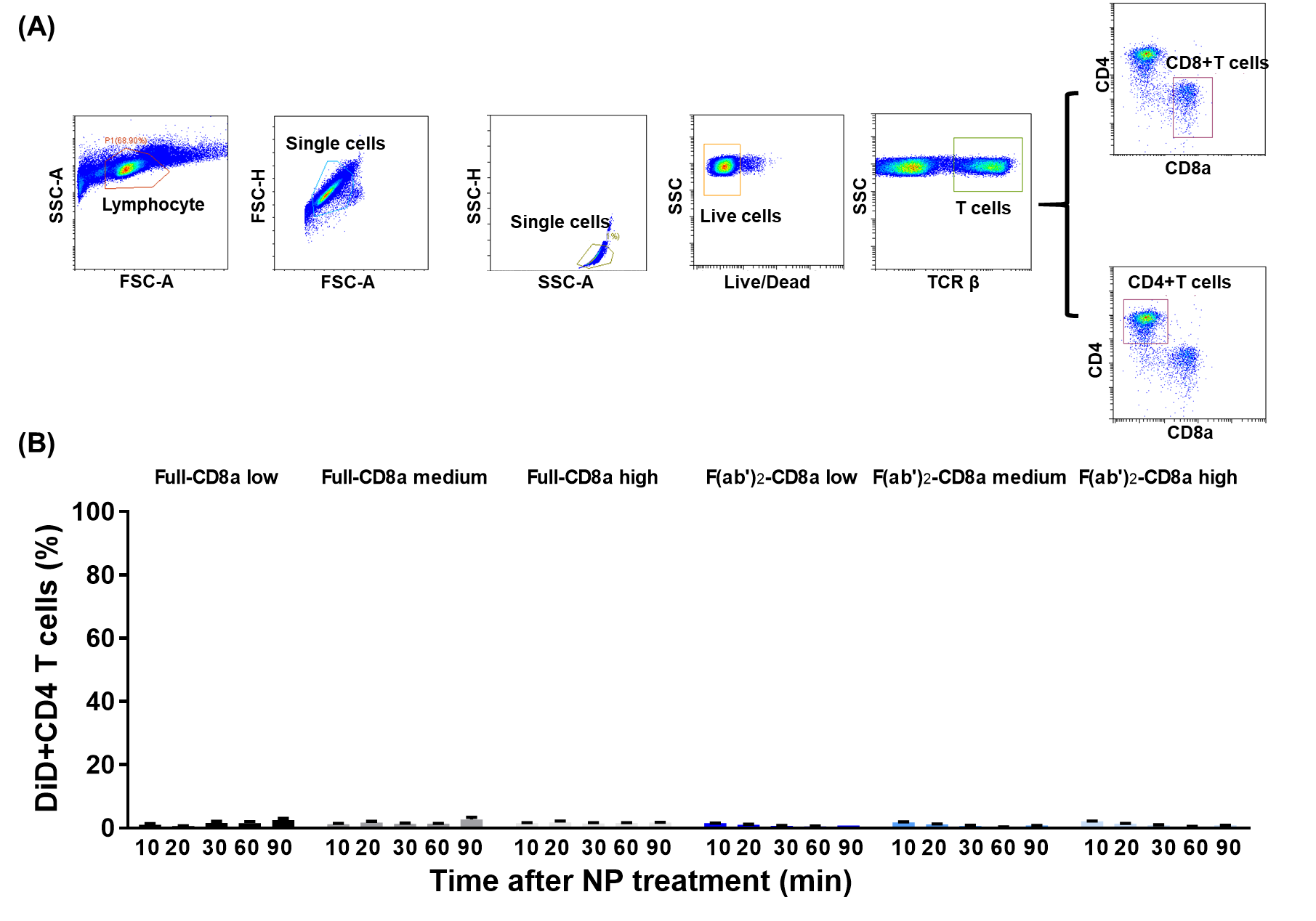


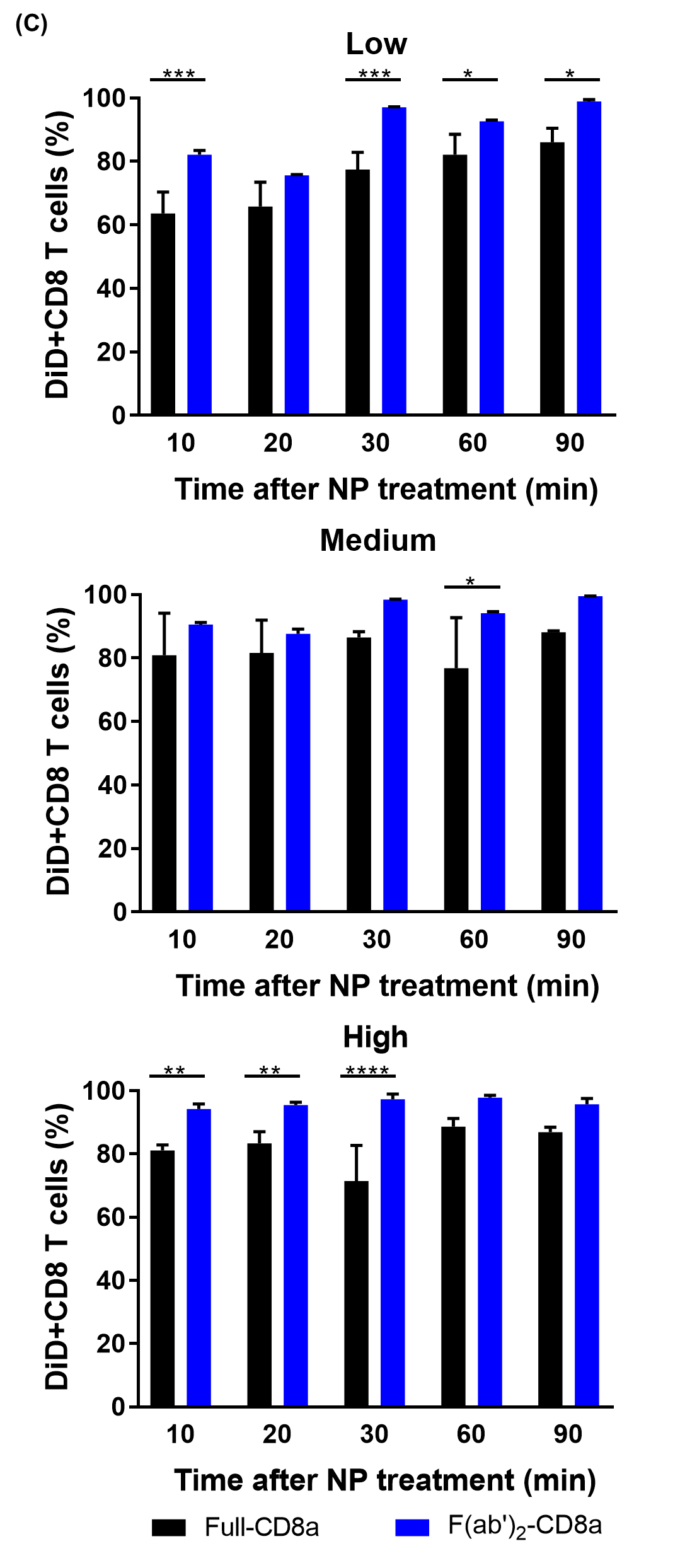


Figure S4. T cell targeting efficiency of the CD8a-conjugated nanoparticles (NPs) *in vitro*. (A) Gating strategy of the CD4 and CD8 + T cells through flow cytometry, (B) The DiD signal observed in the CD4 T cells to confirm the minimal non-specific binding of the anti-CD8a conjugated NPs at different instants, and (C) Comparison between the populations of the DiD-expressing CD8 T cells induced by 20 μg of the full-CD8a NPs and f(ab’)_2_-CD8a NPs at multiple instants. Data were presented as the mean ± SD (n = 3, **P* = 0.0332, ***P* = 0.0021, ****P* = 0.0002, *****P* < 0.0001).
